# Time trends in the use of field-substitution in the Belgian health interview survey

**DOI:** 10.1186/s13690-022-00982-4

**Published:** 2022-11-09

**Authors:** Stefaan Demarest, Geert Molenberghs, Finaba Berete, Rana Charafeddine, Herman Van Oyen, Guido Van Hal

**Affiliations:** 1 Department of Epidemiology and public health, Sciensano, Rue Juliette Wytsmanstraat 14, 1050 Brussels, Belgium; 2 L-Biostat, U Hasselt & KU Leuven, Leuven, Belgium; 3 b Faculty of Medicine and Health Sciences, University of Antwerp, Antwerp, Belgium; 4 Department of Public Health and Primary Care, Ugent, Ghent, Belgium

**Keywords:** Health surveys, Data-collection, Field-substitution

## Abstract

**Background:**

Matched field-substitution has been applied in the Belgian Health Interview Survey (BHIS) since the first round. During data-collection, non-participating households are replaced by substitute households, if needed up to seven times. In this manuscript, the use of field-substitution in the six rounds of BHIS (1997–2018) is assessed. We investigated to what extent field-substitution contributes to obtaining the requested net-sample size and whether this has evolved throughout the successive BHIS’s.

**Methods:**

Harmonized para-data gathered throughout de data-collection phases are used to define the final participation status of all households that could be contacted for participation to the survey. The share of the substituted households was calculated and possible trends in the use of field-substitution throughout the successive surveys was assessed using logistic regression. Finally, it was examined whether the application of field-substitution changed in terms of the position of the participating household in the clusters, using the ESTIMATE statement in the SAS procedure NLMIXED.

**Results:**

Overall, four in ten participating households are substitute households. This proportion remains rather similar over the surveys. The probability of participating according to the position of the household within the cluster is evidently much higher in households at the first position of initial selected clusters. Over the survey-years, the share of participating household derived from substitute clusters in the total number of participating households has slightly increased.

**Conclusion:**

Field-substitution in BHIS plays a very substantial role in obtaining the requested net sample both in size and composition. Field-substitution, as applied in BHIS might inspire scientists to consider it when developing their surveys.

**Supplementary Information:**

The online version contains supplementary material available at 10.1186/s13690-022-00982-4.

## Background

As all in-person surveys, the Belgian Health Interview Survey (BHIS) has to deal with unit non-response. In BHIS, a probability based field-substitution of households, a technique in which non-participating households are replaced (substituted) during the data-collection phase, has been adopted as a means of obtaining a representative net-sample at regional level, as requested by the commissioners of the survey and to ensure that this net-sample is an as unbiased as possible reflection of the gross sample with respect to the age- and household size distribution of the gross sample [1–3]. Literature on field-substitution is scarce and often negative towards its use. The main criticism can be summarized as follows: by using field-substitution, the net sample might deviate from a probability sample and become a convenience sample that over represents easy-to-contact and compliant units, efforts to obtain interviews from the originally selected units might be reduced and field- substitution extends the data-collection period [4–10]. In prominent international surveys, like the International Social Survey Program (ISSP) and the European Health Interview Survey (EHIS), the use of substitution is discouraged. In the European Social Survey (ESS), substitution of nonresponding, non-contactable or non-accessible sampling units is simply not permitted [11]. Yet, in the predominantly negative attitude towards the use of field-substitution reference is made mainly to very basic permissive applications of field-substitution in which, for example, interviewers limit their efforts to contact initially selected units, freely choose substitution units in case of non-participation, hardly any procedures exist to contact units, …

Based on the work of Vehovar [10] and Lynn [6], Smith (2007) proposes a strict protocol to be used in field-substitution to overcome the presumed shortcomings of applying this technique: (a) field-substitution should only be allowed after extensively working initially selected units, (b) strict procedures must exist for when substitution may be used, (c) close supervision is needed to check that interviewers are making the required efforts and follow the protocol, (d) substitute cases are to be chosen at random within strata, (e) when possible, initial and potential substitute cases have to be predefined as part of the master sample design and are matched on several variables based on information from a population register or other sampling frame and (f) the proportion of substitute-units is limited [12]. However, to minimize selection bias, even if strict probabiltiy procedures are applied in field-substitution, data derived from substitutes are apparently considered as ‘second best’ data and their share should be minimized. According to the leading American Association for Public Opinion Research (AAPOR), substitute cases must be marked as such in the dataset - or even stored in a separate dataset - and the substitution procedure must be detailed [13].

For the BHIS 2013, we previously showed that by using field-substitution, the requested net-sample size was obtained, and that its composition reflected the composition of the gross-sample on the requested criteria, without important distorting effects on the estimates [3]. In the current manuscript, the use of field-substitution in the six rounds of BHIS (1997–2018) is assessed based on the para-data stored for each survey. In reporting the results of BHIS, no distinction was made whether the data were based on information provided by initial selected household or by substitute households. In order to align with the recommendation of AAPOR, we investigated to what extent participating households were initial selected or substitute households and whether this had evolved throughout the successive BHIS’s.

## Methods

### Study design and use of field-substitution in BHIS

The BHIS is commissioned by both federal and regional authorities. As such, the protocol that governs BHIS stipulates that the data collection must yield a predetermined number of face-to-face interviews in each of the three regions in the country (Flemish Region, Brussels Capital Region and Walloon Region) and, consequently, in an overall fixed net-sample size. BHIS is by design a household survey. Households are invited to participate and when a household participates, at most four household members are selected for participation. To build a probabilistic sample of households, a multistage sampling design is applied, including stratification, clustering, systematic sampling and simple random sampling. Stratification is used to ensure the predetermined number of interviews at regional level [1, 14–16].

In both the Flemish and Walloon Regions, provincial stratification is applied, in which the sample size per province is proportional to the population size of the province. The Brussels Capital Region is treated as one province. Within each province, municipalities (Primary Sampling Units, PSUs) are selected through a systematic sampling method with a selection probability proportional to their size. In each selected municipality, systematic sampling (based on statistical sector, age and household size) is used to select households (Secondary Sampling Units, SSUs) using the National Register as the sampling frame. The number of selected households corresponds to fifty individuals who have to be interviewed throughout a 1 year period of data collection (spread over four quarters, so on average 12½ per quarter). Finally, at most four individuals (Tertiary Sampling Units, TSU’s) are invited for participation within each selected household: by definition the household reference person and as appropriate the partner and probabilistic based on a birthday rule two additional household members [1, 14, 17].

Field substitution in the BHIS is applied at the level of the of SSUs: after having hierarchically ordered the households according to statistical sector (smallest territorial unit, comparable with a ward or a district), household size (size 1, 2, 3, and 4+) and age-group (in 7 categories) of the households’ reference person (the person who represents the household in relation with the authorities), per selected household three matched (substitute households) are generated. The so-resulting group of matched quadruples households is called a cluster of households. To account for the uncertainty that the number of interviews cannot be determined with certainty (given possible within household refusal or differences between the administrative and the ‘real-life’ composition of the households selected for participation) for each initial selected cluster a substitute cluster was created that, although composed in exactly the same way, was not matched with the initial cluster for household size and age of the household reference person (1,16,20).

The notion of ‘clusters’ is key for the BHIS substitution procedure. After the clusters have been created, vertical scrambling of clusters and horizontal scrambling of households within the clusters is applied in order to eliminate any kind of systematic approach. At the start of each trimester, all households at the first position of the initial clusters are activated; that is, to all these households an invitation is sent to participate in the BHIS. At the same moment, the interviewers receive the addresses of the households invited for participation, so they can start contacting the households. Each contact attempt has to be registered (day/hour of contact, mode of contact, result of contact) and for each household at least five contact attempts have to be made. In case of frame errors (address does not exist, the residence is clearly abandoned, the household has moved or the address is the address of a prison, a monastery or a psychiatric institution) the household – and all its members - will be labelled as non-eligible and will be substituted. In case the selected households reside at the address and all contact attempts turn out to be fruitless, the household can be labelled as ‘non-contactable’. When a household is contacted, it can refuse to participate (‘refusal’) or it can participate (‘participation’). The latter can only be confirmed once the results of the interview(−s) are uploaded in the central management system. In order to discourage substitution, interviewers are only rewarded for successful participating households. In case a household turns out to be non-contactable or refuses to participate, the central secretariat of BHIS substitutes the household by a household positioned in consecutive order and these households are invited to participate in the BHIS. The procedure to be applied by the interviewers remains unchanged. If necessary, substitution will be applied until the cluster is exhausted (all households of the cluster turn out to be non-contactable or refusing households). Then a substitute cluster will be activated; the household positioned at the first place in the substitute cluster will be invited to participate and will be substituted, if necessary. In the (rare) cases that also the substitute cluster is exhausted, the process ends. The data-collection phase is set at one calendar year, split into four trimesters. Due to field-substitution, data collection is spread throughout the trimesters. If necessary, interviews scheduled for a trimester can be realized in the next trimester(−s). At the end of the year, data collection is interrupted but interviewers have to finalise the interviews in households for which they have appointments. In case the fixed number of interviews in a region is realized before the end of the year, data-collection stops prior to the end of the year [16, 18].

From the first round of BHIS (1997), till the BHIS 2008, data-collection was organized by Sciensano using a interviewers pool specifically created for the survey. From the BHIS 2013 on, data-collection was subcontracted to Statbel, the Belgian Statistical Office and integrated in the data-collection approach used in its other surveys (Labor Force Survey, Statistics on Income and Living Conditions, …).

### Data

The number of households invited for participation in the BHIS’s differs according to the survey year, given provincial oversampling (2004, 2008, and 2013), oversampling of the elderly age groups (2004, 2008), oversampling of the German Community (2018) and the protocol-based increase of the base-sample for the Flemish Region (2018). For all households invited for participation in the BHIS’s, info on the survey-year, the region, the age group of the households’ reference person and the household size was derived from the NR used as the sampling frame for BHIS. Initial selected clusters and substitute clusters were identified after vertical scrambling, the position of the households was defined after horizontal scrambling within the (substitute) clusters. The final participation status of the households was derived from the fieldwork management system and linked with NR data. The consistency of the statuses was checked prior to the analysis of the data (e.g. within a cluster a participating household was a household at the first position or could only be preceded by (a) non-participating household).

### Analysis

First, the evolution in non-participation rates and field substitution rates will be assessed throughout the survey-years, using an analytic approach inspired by Baldissera et al. [19]: for each of the surveys, initial selected households (INI_Survey_) lead to participating households (PINI_Survey_) and non-participating households (NPRINI_Survey_) (Table 1). Non-participating households are substituted, if needed, several times, by substitute households (SUB_Survey_). These substitute households can either participate (PSUB_Survey_) or also turn into non-participating households (NPRSUB_Survey_).

**Table 1:** Description of non-response, substitution, participation rates and substitution rates in the Belgian Health Interview Survey

Households selected for particiation	Initial selected	Substitute	Total	Substitution rates
**Participating households**	PINI_Survey_	PSUB_Survey_	PTOT_Survey_	PSUB_Survey_/PTOT_Survey_
**Non-participating households**	NPRINI_Survey_	NPRSUB_Survey_	NPRTOT_Survey_	NPRSUBSurvey/NPRTOT_Survey_
**Total**	INI_Survey_	SUB_Survey_	TOT_Survey_	SUBSurvey/TOT_Survey_
**Participation rates**	NPRINI_Survey_/INI_Survey_	NPRSUB_Survey_/SUB_Survey_	NPRTOT_Survey_/TOT_Survey_	

PINI_Survey_: participating households among the initial selected households

NPRINI_Survey_: non-participating households among the initial selected households

INI_Survey_: total initial selected households

NPRINI_Survey_/INI_Survey_: proportion non-participating initial selected housholds

PSUB_Survey_: participating households aming substitute households

NPRSUB_Survey_: non-participating households among substitute households

SUB_Survey_: total substitute households

NPRSUB_Survey_/SUB_Survey_: proportion non-participating substitute households

PTOT_Survey_: total participating households

NPRTOT_Survey_: total non-participating households

TOT_Survey_: total households invited for participation

NPRTOT_Survey_/TOT_Survey_: proportion non-participating households

PSUB_Survey_/PTOT_Survey_: proportion participating substitute household among all households invited for participation

NPRSUB_Survey_/NPRTOT_Survey_: proportion non-participating substitute households among all non-participating households

SUB_Survey_/TOT_Survey_: proportion substitute households among all households invited for participation

Based on this, the non-participation rates among initial selected households (NPRIN_Survey_/INI_Survey_), among substitute households (NPRSUB_Survey_/SUB_Survey_) and among all households invited for participation (NPRTOT_Survey_/TOT_Survey_) can be calculated for every survey-year. The substitution rates are expressed as the share of participating substitute households in all participating households (PSUB_Survey_/PTOT_Survey_), the share of non-participating substitute housholds in all non-participating households (NPRSUB_Survey_/NPRTOT_Survey_) and the share of all subsitute households invited for participation in all households invited for participation (SUB_Survey_/TOT_Survey_).

Second, logistic regression is used to assess time trends in substitution, taking into account differences between the successive surveys in terms of age group of the household’s reference person, household size and trimester for which the initial households were invited to participate. Since in BHIS predefined numbers of interviews had to be realized at regional level, the analysis was done stratified by region.

Third, it was examined whether the application of field-substitution changed in terms of the position of the participating household in the clusters (i.e., the probability of finding a participating household in a given survey year at one position in the cluster versus the probability of finding a participating household in another survey year at the same position). These probabilities were calculated from the ESTIMATE statement in the SAS procedure NLMIXED.

## Results

The percentage non-participants among initially selected households (NPRINI_Survey_) decreased between the first BHIS (1997) and the BHIS 2004 (from 35.7 to 26.8%) (Table 2). This percentage increased substantially in the BHIS 2008 (38.0%), but dropped thereafter to reach 30.9% in the BHIS 2018. For every survey year, the percentage of non-participating substitute households (NPRSUB_Survey_) is higher than the percentage found for initially selected households (NPRINI_Survey_). Consequently, the overall non-participation percentages (NP_Survey_) are higher after than before applying substitution.

**Table 2:** Non-response, substitution, participating rates and substitution rates in the successive waves of the Belgian Health Interview Surveys, 1997–2018

	NPRINI_**Survey**_/INI_**Survey**_ (%)	NPRSUB_**Survey**_/SUB_**Survey**_ (%)	NPRTOT_**Survey**_/TOT_**Survey**_ (%)	PSUB_**Survey**_/PTOT_**survey**_ (%)	NPRSUB_**Survey**_/NPRTOT_**Survey**_ (%)	SUB_**Survey**_/TOT_**Survey**_ (%)
**BHIS 1997**	52.7	59.5	56.0	45.3	52.3	49.2
**BHIS 2001**	46.9	56.1	50.8	38.1	47.1	42.7
**BHIS 2004**	42.3	54.7	47.6	37.0	49.2	42.8
**BHIS 2008**	55.1	63.7	59.2	42.6	51.5	47.9
**BHIS 2013**	45.1	51.1	47.7	41.4	47.3	44.2
**BHIS 2018**	47.2	49.7	48.3	42.0	44.4	43.2

NPRINISurvey/INIsurvey: % of non-participating households among all initial selected housholds

NPRSUBsurvey/SUBsurvey): % of non-participating households among all substitute households

NPRTOTsurvey/TOTsurvey: % of non-participating households among all households

PSUBsurvey/PTOTsurvey: % of participating substitute households among all participating households

NPRSUBsurvey/NPRTOTsurvey: % of non-participating substitute households among all non-participating households

SUBsurvey/TOTsurvey: % of substitute households among all households invited for participation

The logistic regression shows that in the survey-years following the BHIS 1997, the odds of being a participating substitution household decreased and this in all regions (Table 3). In more recent survey-years (BHIS 2013, BHIS 2018), no significant differences in odds compared to the BHIS 1997 can be observed.

**Table 3:** Odd’s ratios (+ 95% CI’s) of being a participating substitute household in the successive waves of Belgian Health Interview, by region, 1997–2018

		Flemish Region		Brussels Region		Walloon Region	
Year	1997	Reference		Reference		Reference	
	2001	0.78	(0.68–0.90)	0.67	(0.58–0.78)	0.60	(0.52–0.68)
	2004	0.74	(0.65–0.85)	0.71	(0.62–0.82)	0.52	(0.46–0.60)
	2008	0.80	(0.69–0.92)	0.83	(0.72–0.95)	0.61	(0.53–0.69)
	2013	0.97	(0.84–1.12)	0.97	(0.84–1.13)	0.77	(0.68–0.89)
	2018	1.00	(0.87–1.16)	0.98	(0.86–1.12)	0.88	(0.77–1.00)
Age-group	0–24 years	Reference		Reference		Reference	
	25–44 years	1.03	(0.80–1.31)	1.17	(0.94–1.46)	0.82	(0.67–1.02)
	45–64 years	1.22	(0.95–1.56)	1.30	(1.04–1.62)	0.92	(0.75–1.14)
	65+ years	1.19	(0.93–1.52)	1.41	(1.13–1.76)	1.05	(0.85–1.29)
Trimester	First trimester	Reference		Reference		Reference	
	Second trimester	0.96	(0.86–1.08)	1.10	(0.99–1.24)	0.95	(0.86–1.05)
	Third trimester	1.05	(0.94–1.17)	0.99	(0.88–1.10)	0.89	(0.80–0.99)
	Fourth trimester	0.83	(0.74–0.93)	0.81	(0.72–0.90)	0.71	(0.64–0.79)

In both the Flemish and Walloon Regions, the odds of being a participating substitution household is not related to the age group of the household’s reference person. In the Brussels Capital Region, the odds of being a substitute household is higher in households with an older reference person. Evidently, in all regions the odds of being a participating substitute household is significantly lower in households scheduled for participation in the fourth (last) trimester of data-collection. While the process of field-substitution was allowed to cross trimester-borders, data collection had to stop at the end of the last trimester.

Several logistic models for ordinal data were tested to assess the probability of participating to the survey, according to the place of the household in the clusters: proportional odds – linear/unstructured in survey round without covariates; non-proportional odds – unstructured in survey round without and with covariates and split by covariates (see supplementary material 1 for the models building). Based on the models’ deviances, the model with fully separate analyses stratified by region (Model 4) provides a better fit and was therefore retained as the final model.

**Fig. 1:**
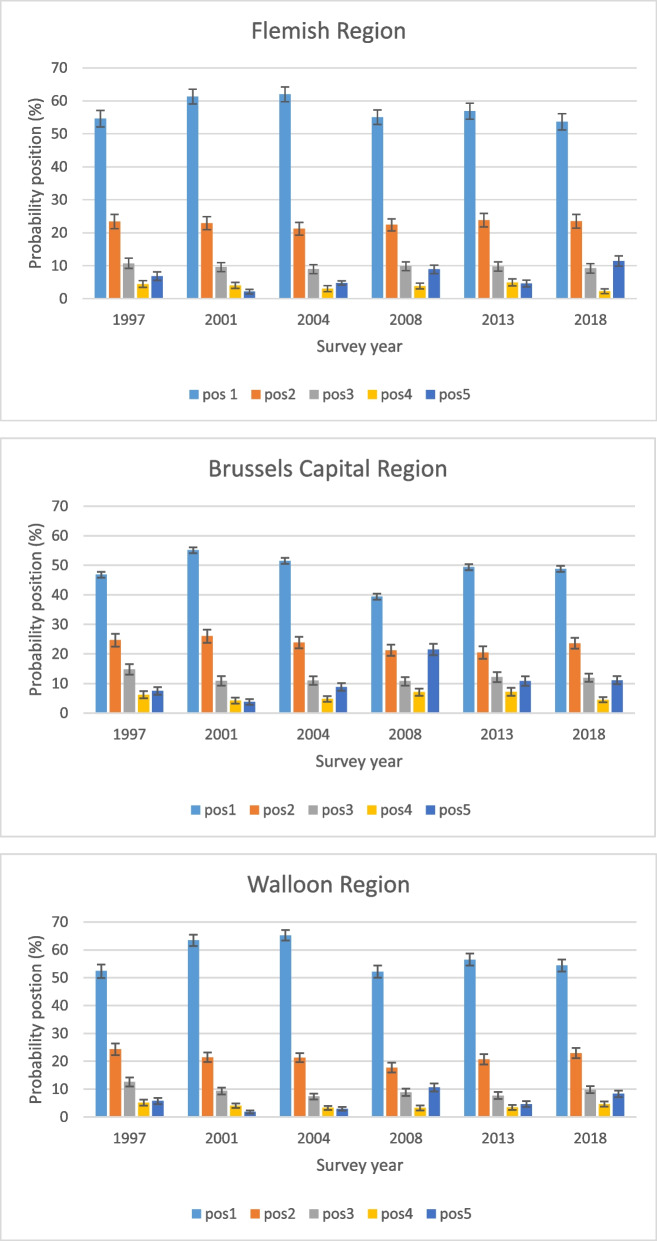
Probabilities of participating to the Belgian Health Interview Survey according the place of the invited households in the clusters, by region, 1997–2018

The probability of participation depends evidently on the position of the household within the cluster. In the Flemish Region, in the BHIS 1997, the probability of finding a participating household at the first position (an initial selected household) is 54.6%, the probability of finding such household in a substitute cluster (position 5 – refers to participating household at any position in the substitute cluster) is 6.8% (Fig. 1 – corresponding table in supplementary material 2). Over the survey years, the pattern is very similar between the Flemish and Walloon Regions, but different for the Brussels Capital Region. In the latter, the probability of finding a participating household at the first position is lower compared to both other regions. In the Brussels Capital Region, from the BHIS 2008 on, the probability of finding a participating initial selected household (positon 1) is even lower than 50%.

For both the BHIS 2001 and BHIS 2004, the probability of finding a participating initial selected household steadily increased, making the use of field-substitution somewhat less important to achieve the predefined number of participants (defined at individual level for each of the regions).

The data collection in the context of the BHIS 2008, is an outlier compared to the other BHIS’s, with relative low probabilities of finding initial participation households (in the Brussels Capital Region only 39.4%) and, consequently, higher probabilities of finding participating households at further positions in the cluster. For the Brussels Capital Region, in the BHIS 2008, not less than 21.5% of all participating households are part of a substitution cluster.

The pattern of participating to the BHIS 2013 and BHIS 2018 resembles the one found for the first three rounds of BHIS. Yet, the probability of requiring participating households from substitute clusters increases until around 10%.

When comparing the pattern of participation between the BHIS 1997 and the BHIS 2018, the probabilities of finding participating households at the different positions in the clusters do not significantly change, except for the households belonging to substitute clusters (position 5).

## Discussion

While preparing the first BHIS, it was opted to apply a probability field substitution procedure during data-collection. This was considered as the best guarantee to anticipate non-participation and to obtain the net sample size as requested by the commissioners of the survey both in terms of size and composition and this for each of the regions [3, 16]. The managed field substitution during the field work followed the complex stratified, clustered, and systematic sampling design by replacing non-participating households with another household selected on a probability basis. A transparent and strict substitution scheme was developed in which interviewers had to document every contact attempt before declaring a household invited for participation as a non-contactable or refusing household and in which substituting non-participating households is the prerogative of the central secretariat.

Although substitution of non-participating households was conceived as an exception, the assessment of the use of field-substitution throughout the successive rounds of BHIS shows that almost half of the participating households are substitute-households. In the survey-years following the first BHIS, the share of substitute-households decreased. In recent survey-years this share increased again. Field-substitution, as applied in BHIS is thus not a ‘corrective’ strategy to deal with non-participation, but turned out to be an indispensable tool in obtaining the predetermined net-sample size. To our knowledge, no reference exists on the limitations in using field-substitution, but in the few studies that used and reported on the use of field-substitution, the substitution rate ranges from 27% [19] to 35% [20] – substantially lower than the substitute rates found in BHIS.

For all rounds of the BHIS, the non-participation rate is higher among substitute-households than among initially selected households. This indicates that field-substitution is more prevalent among ‘hard to reach’ households. Since the technique of matched substitution is applied, it might be that non-participating ‘hard to reach’ households are substituted by similar households, yielding in higher non-participation rates.

The need to use field-substitution decreased between the BHIS 1997 and the BHIS 2013 and then increased again till the level of the BHIS 1997. In the BHIS 2013 the data-collection in the context of the BHIS was outsourced to Statbel and framed into its other data-collections. Although the field-substitution procedure remained unchanged, it might be that the interviewers – used to work for other Statbel surveys - tended to label households as non-participating households somewhat sooner than in the previous rounds of the BHIS.

In the last (fourth) trimester of every BHIS, the odds of being a substitute household is substantially lower compared the other trimesters. This is a normal event: after the fourth trimester data-collection has to be terminated leaving less time to substitute non-participating households.

The pattern of participating in the BHIS is different in the Brussels Capital Region compared with both the Flemish and Walloon Regions. In the metropolitan Brussels Capital Region, overall participation to the survey is lower; hence the need for field-substitution is higher. Lower participation in this region is due to a high number of households that could not be contacted, since they moved between the moment of sampling and the first contact-attempts by the interviewers. Also higher refusal rates, due to language problems and the inability for interviewers to have a face-to-face contact with the households (only by intercom at the door.

in large apartments) contributed to a lower participation rate. Lower participation rates in BHIS is not specific for the Brussels Capital Region; analysis of data from the Health Survey for England 2006 and the Boost Survey for London showed lower household participation rates in London and other metropolitan areas than elsewhere in England and was associated with a younger demographic, higher migrant populations, and therefore more non-native speakers, and greater deprivation, associated with lower literacy levels [21].

Data-collection for the BHIS 2008 was, compared to the other rounds of BHIS, an outlier. This was mainly related to privacy issues in using the data of the National Register. While the samples for the first and second trimester were composed as scheduled, the start of data-collection had to be delayed until May 2008. As a consequence, partially outdated samples had to be used, yielding in high percentages of households selected for participating that had moved in the mean while.

It can be argued that, given the rather stable level of non-participation and consequently the need for field-substitution in BHIS, inflating the gross-sample accordingly would make field-substitution redundant. This alternative entails some considerable set-backs: (a) it requires the deployment of a much larger group of interviewers (while it is very difficult to find interviewers), (b) it would yield a concentration of interviews close to the start of each trimester (while, due to field-substitution, a spread of interviewers throughout the year of data-collection is achieved) and (c) if offers no guarantees whatsoever that the net-sample resembles the composition of the gross-sample in terms of the selection criteria (while this is one of the major strengths of applying field-substitution).

In order to assess the possible impact of field-substitution on the (health) estimates, it might worthwhile to provide in the database information whether a participating household is an initial selected or a substitute household, as suggested by AAPOR [13]. Yet, it has been shown that field-substitution does not introduce extra bias to the estimates [3, 19]. On the contrary, it might be assumed that field-substitution leads to bias reduction. Indeed, in the extreme case that the matching variables (statistical sector, household size, age group of the reference person) would correlate perfectly with the health estimates, bias would be completely avoided. In case the correlation is close to null, applying field-substitution would have no impact in neither sense. In any case, applying substitution is preferable compared to a ‘do nothing’ or to a sampling inflation strategy to cope with non-participation. Although it has to be admitted that applying field-substitution does not vanish bias - a residual bias remains – the bias without the use of field-substitution would be (substantially) higher.

This study is, to our knowledge, the first one that assesses the evolution in the use of field-substitution throughout successive survey years in which this technique was applied. It showed that field-substitution is indispensable in order to obtain the requested sample size, and assures that the net-sample reflects the composition of the gross-sample according to the selection criteria.

It has to be admitted that face-to-face surveys, like BHIS, rely heavily on the interviewers collecting the data. Although all interviewers were instructed in detail how to conduct the survey (contacting the household, convincing the household to participate, completing the questionnaire, …) uncertainties remain on how these instructions were applied. This is especially the case when labelling households as non-contactable or refusing. Interviewers might easily invent contact-attempts to fulfill the minimal required contact attempts or deceptively label a household as non-contactable or refusing to participate. Assessing the correct use of field-substitution is as such hindered by possible shortcomings at the source of is application. Under Belgian privacy law, it is forbidden to re-approach households that did not participate, making an in-depth inquiry of the nature of non-participation impossible.

## Conclusion

Applying field-substitution will not solve the problem of non-participation, inherently to surveys. Yet it merits a profound analysis of its advantages and disadvantages when developing a survey design. A strict centrally managed procedure limits the possible impact of interviewers on the field-substitution process, hereby responding to the classic criticism towards applying this technique. An undisputable advantage of applying field-substitution is that is assures the realization of the requested net sample size and guarantees that the net-sample composition – in terms of the matching criteria – coincides with the composition of the gross sample. Field-substitution, as applied in BHIS might inspire scientists to consider it when developing their surveys.

## Supplementary Information

**Additional file 1: Supplementary material 1.** Mathematical specifications of the models. **Supplementary material 2.** Probabilities of participating to BHIS according the place of the invited households in the clusters, BHIS 1997 – BHIS 2018.

## Data Availability

The data of this study are available from the corresponding author upon reasonable request.
