# Supplementary material for: Time trends in the use of field-substitution in the Belgian health interview survey
Source: Arch Public Health. 2022 Nov 9;80:229. doi: 10.1186/s13690-022-00982-4 (PMC9644564; doi:10.1186/s13690-022-00982-4)
Supplement: Supplementary file 1 — Additional file 1: Supplementary material 1. Mathematical specifications of the models. Supplementary material 2. Probabilities of participating to BHIS according the place of the invited households in the clusters, BHIS 1997 – BHIS 2018. [file 13690_2022_982_MOESM1_ESM.docx]

**Supplementary material 1:**

**Mathematical specifications of the models :**

Write the logit of the probability that the household sequence number S_y_ in wave ($y$ = 0,...,5) is equal to $k$ ($k$ = 1,...,5) as:

$$P\left( S_{y}=k \right)=\frac{1}{1+e^{-\eta k}}- \frac{1}{1+e^{-\eta k-1}}$$

and parameterize the logit functions η_k_ as follows:

$$\eta_{0}= -\infty,$$

$$\eta_{k}= \alpha_{k}-\sum_{l=0}^{k} \beta_{lk}I\left( l=y \right), \left( k=1,\ldots,4 \right),$$

$$\eta_{5}= -\infty.$$

Should the parameters $\beta_{lk}$be independent of $k$, then a proportional-odds model would follow. The current model, therefore, is non-proportional-odds. Note that the logit $\eta_{0}$ corresponds to probability zero upon expit transformation, as it should, because the probability that the substitution number is 0 is non-existent. Likewise, the probability that the substitution number is less than or equal to 5 is 1, so $\eta_{5}$ corresponds to a probability of 1, upon expit transformation. All parameters are estimated for each region separately.

The models are fitted with the NLMIXED procedure in SAS, even though there are no random effects, but because the procedure offers a convenient and flexible way of specifying generalized linear models that are somewhat less conventional, such as a non-proportional-odds logistic regression.

**The SAS code.**

**proc** **nlmixed** data=hulp;

title 'Model 4: HIS Substitution - non-proportional odds - unstructured in survey wave';

title2 'By region';

by region;

parms int1=**0** int2=**1** int3=**2** int4=**2.5**

beta11=-**0.3** beta21=-**0.3** beta31=**0.25** beta41=-**0.06** beta51=**0.08**

beta12=-**0.3** beta22=-**0.3** beta32=**0.25** beta42=-**0.06** beta52=**0.08**

beta13=-**0.3** beta23=-**0.3** beta33=**0.25** beta43=-**0.06** beta53=**0.08**

beta14=-**0.3** beta24=-**0.3** beta34=**0.25** beta44=-**0.06** beta54=**0.08**

;

beta01=**0**;

beta02=**0**;

beta03=**0**;

beta04=**0**;

eta1 = beta01*(survey=**0**)+beta11*(survey=**1**)+beta21*(survey=**2**)+beta31*(survey=**3**)+beta41*(survey=**4**)+beta51*(survey=**5**);

eta2 = beta02*(survey=**0**)+beta12*(survey=**1**)+beta22*(survey=**2**)+beta32*(survey=**3**)+beta42*(survey=**4**)+beta52*(survey=**5**);

eta3 = beta03*(survey=**0**)+beta13*(survey=**1**)+beta23*(survey=**2**)+beta33*(survey=**3**)+beta43*(survey=**4**)+beta53*(survey=**5**);

eta4 = beta04*(survey=**0**)+beta14*(survey=**1**)+beta24*(survey=**2**)+beta34*(survey=**3**)+beta44*(survey=**4**)+beta54*(survey=**5**);

if ininom=**1** then z = **1**/(**1**+exp(-(int1-eta1)));

else if ininom=**2** then z = **1**/(**1**+exp(-(int2-eta2))) - **1**/(**1**+exp(-(int1-eta1)));

else if ininom=**3** then z = **1**/(**1**+exp(-(int3-eta3))) - **1**/(**1**+exp(-(int2-eta2)));

else if ininom=**4** then z = **1**/(**1**+exp(-(int4-eta4))) - **1**/(**1**+exp(-(int3-eta3)));

else z = **1** - **1**/(**1**+exp(-(int4-eta4)));

if z > **1e-8** then ll = log(z);

else ll = -**1e100**;

model ininom ~ general(ll);

estimate 'probability position 1 in 1997' **1**/(**1**+exp(-(int1-beta01)));

estimate 'probability position 2 in 1997' **1**/(**1**+exp(-(int2-beta02))) - **1**/(**1**+exp(-(int1-beta01)));

estimate 'probability position 3 in 1997' **1**/(**1**+exp(-(int3-beta03))) - **1**/(**1**+exp(-(int2-beta02)));

estimate 'probability position 4 in 1997' **1**/(**1**+exp(-(int4-beta04))) - **1**/(**1**+exp(-(int3-beta03)));

estimate 'probability position 5 in 1997' **1** - **1**/(**1**+exp(-(int4-beta04)));

estimate 'probability position 1 in 2001' **1**/(**1**+exp(-(int1-beta11)));

estimate 'probability position 2 in 2001' **1**/(**1**+exp(-(int2-beta12))) - **1**/(**1**+exp(-(int1-beta11)));

estimate 'probability position 3 in 2001' **1**/(**1**+exp(-(int3-beta13))) - **1**/(**1**+exp(-(int2-beta12)));

estimate 'probability position 4 in 2001' **1**/(**1**+exp(-(int4-beta14))) - **1**/(**1**+exp(-(int3-beta13)));

estimate 'probability position 5 in 2001' **1** - **1**/(**1**+exp(-(int4-beta14)));

estimate 'probability position 1 in 2004' **1**/(**1**+exp(-(int1-beta21)));

estimate 'probability position 2 in 2004' **1**/(**1**+exp(-(int2-beta22))) - **1**/(**1**+exp(-(int1-beta21)));

estimate 'probability position 3 in 2004' **1**/(**1**+exp(-(int3-beta23))) - **1**/(**1**+exp(-(int2-beta22)));

estimate 'probability position 4 in 2004' **1**/(**1**+exp(-(int4-beta24))) - **1**/(**1**+exp(-(int3-beta23)));

estimate 'probability position 5 in 2004' **1** - **1**/(**1**+exp(-(int4-beta24)));

estimate 'probability position 1 in 2008' **1**/(**1**+exp(-(int1-beta31)));

estimate 'probability position 2 in 2008' **1**/(**1**+exp(-(int2-beta32))) - **1**/(**1**+exp(-(int1-beta31)));

estimate 'probability position 3 in 2008' **1**/(**1**+exp(-(int3-beta33))) - **1**/(**1**+exp(-(int2-beta32)));

estimate 'probability position 4 in 2008' **1**/(**1**+exp(-(int4-beta34))) - **1**/(**1**+exp(-(int3-beta33)));

estimate 'probability position 5 in 2008' **1** - **1**/(**1**+exp(-(int4-beta34)));

estimate 'probability position 1 in 2013' **1**/(**1**+exp(-(int1-beta41)));

estimate 'probability position 2 in 2013' **1**/(**1**+exp(-(int2-beta42))) - **1**/(**1**+exp(-(int1-beta41)));

estimate 'probability position 3 in 2013' **1**/(**1**+exp(-(int3-beta43))) - **1**/(**1**+exp(-(int2-beta42)));

estimate 'probability position 4 in 2013' **1**/(**1**+exp(-(int4-beta44))) - **1**/(**1**+exp(-(int3-beta43)));

estimate 'probability position 5 in 2013' **1** - **1**/(**1**+exp(-(int4-beta44)));

estimate 'probability position 1 in 2018' **1**/(**1**+exp(-(int1-beta51)));

estimate 'probability position 2 in 2018' **1**/(**1**+exp(-(int2-beta52))) - **1**/(**1**+exp(-(int1-beta51)));

estimate 'probability position 3 in 2018' **1**/(**1**+exp(-(int3-beta53))) - **1**/(**1**+exp(-(int2-beta52)));

estimate 'probability position 4 in 2018' **1**/(**1**+exp(-(int4-beta54))) - **1**/(**1**+exp(-(int3-beta53)));

estimate 'probability position 5 in 2018' **1** - **1**/(**1**+exp(-(int4-beta54)));

estimate 'difference in probs for position 1 in 2018 versus 1997'

**1**/(**1**+exp(-(int1-beta51))) - **1**/(**1**+exp(-(int1-beta01)));

estimate 'difference in probs for position 2 in 2018 versus 1997'

**1**/(**1**+exp(-(int2-beta52))) - **1**/(**1**+exp(-(int1-beta51))) - **1**/(**1**+exp(-(int2-beta02))) + **1**/(**1**+exp(-(int1-beta01)));

estimate 'difference in probs for position 3 in 2018 versus 1997'

**1**/(**1**+exp(-(int3-beta53))) - **1**/(**1**+exp(-(int2-beta52))) - **1**/(**1**+exp(-(int3-beta03))) + **1**/(**1**+exp(-(int2-beta02)));

estimate 'difference in probs for position 4 in 2018 versus 1997'

**1**/(**1**+exp(-(int4-beta54))) - **1**/(**1**+exp(-(int3-beta53))) - **1**/(**1**+exp(-(int4-beta04))) + **1**/(**1**+exp(-(int3-beta03)));

estimate 'difference in probs for position 5 in 2018 versus 1997'

**1** - **1**/(**1**+exp(-(int4-beta54))) -**1** + **1**/(**1**+exp(-(int4-beta04)));

estimate 'probability position 1 or 2 in 1997' **1**/(**1**+exp(-(int2-beta02)));

estimate 'probability position 2 or 3 or 4 in 1997' **1**/(**1**+exp(-(int4-beta04))) - **1**/(**1**+exp(-(int1-beta01)));

estimate 'difference in probs for position 1 in 2001 versus 1997' **1**/(**1**+exp(-(int1-beta11))) - **1**/(**1**+exp(-(int1-beta01)));

**run**;

**Results tested logistic models for ordinal data to assess the probability of participating to the BHIS survey.**

| **Model** |  | **# parameters** | **-2 log- likelihood** | **Models being compared** | **Deviance** | **Degrees of freedom for comparison** | **p-value** |
| --- | --- | --- | --- | --- | --- | --- | --- |
| 1 | PO, linear in survey | 5 | 81844 |  |  |  |  |
| 2 | PO, unstruct in survey | 9 | 81503 | 1 – 2 | 341 | 4 | <0.0001 |
| 3 | Non-PO, unstruct in survey | 24 | 81340 | 2 – 3 | 163 | 15 | <0.0001 |
|  |  |  |  | 1 – 3 | 504 | 19 | <0.0001 |
| 4 | By region | 72 | 80565 | 3 – 4 | 775 | 48 | <0.0001 |
| 5 | Two region dummies | 26 | 81025 | 3 – 5 | 315 | 2 | <0.0001 |
| 6 | One region dummy (for region 2) | 25 | 81016 | 3 - 6 | 324 | 1 | <0.0001 |
|  |  |  |  | 5 – 6 | 9 | 1 | 0.0027 |
|  |  |  |  | 4 – 5 | 460 | 46 | <0.0001 |
| 7 | By trim | 96 | 80634 | 3 – 7 | 706 | 72 | <0.0001 |
| 8 | Dummies for trim | 27 | 81143 | 3 – 8 | 197 | 3 | <0.0001 |
|  |  |  |  | 7 – 8 | 509 | 69 | <0.0001 |
| 9 | HH size | 25 | 81013 | 3 – 9 | 327 | 1 | <0.0001 |
| 10 | Age4 | 25 | 81090 | 3 – 10 | 250 | 1 | <0.0001 |
| 11 | All covariates | 33 | 80783 | 3 – 11 | 557 | 9 | <0.0001 |

- We first investigate whether proportional odds is sufficient and whether survey wave can be included linearly or via dummies (unstructured). It turns out that the more complex choices of these are necessary, so the baseline model is Model 3: non-proportional odds and unstructured in survey.
- Models 4 – 6 examine the effect of region. The most drastic choice is fully separate analyses per region (Model 4), instead of dummies for region (Models 5 and 6). It turns out that a model by region provides a much better fit.
- In Models 7 and 8, the effect of trimester is examined. Also here, a model by trimester separately works much better.
- Finally, in Model 9 the effect of household size is established and in Model 10 an effect of age (in its categorical form) is shown to be present.
- In Model 11, all covariates (region, trimester, age4, HH size) are simultaneously taken into account. All are significant.

**Supplementary material 2:**

**Probabilities of participating to BHIS according the place of the invited households in the clusters, BHIS 1997 – BHIS 2018**

|  | Flemish Region | | Brussels Region | | Walloon Region | |
| --- | --- | --- | --- | --- | --- | --- |
|  | Prob. | 95% CI | Prob. | 95% CI | Prob. | 95% CI |
| **BHIS 1997** |  |  |  |  |  |  |
| position 1 | 54.6 | (52.1 - 57.1) | 46.8 | (44.4 - 49.3) | 52.4 | (49.9 - 54.8) |
| position 2 | 23.4 | (21.3 - 25.6) | 24.7 | (22.5 - 26.8) | 24.3 | (22.2 - 26.4) |
| position 3 | 10.7 | (9.2 - 12.3) | 14.8 | (13.0 - 16.5) | 12.6 | (11.0 - 14.2) |
| position 4 | 4.4 | (3.4 - 5.5) | 6.2 | (5.0 - 7.4) | 5.1 | (4.0 - 6.2) |
| position 5 | 6.8 | (5.6 - 8.1) | 7.5 | (6.2 - 8.8) | 5.7 | (4.6 - 6.8) |
| **BHIS 2001** |  |  |  |  |  |  |
| position 1 | 61.3 | (59.1 - 63.5) | 55.1 | (52.6 - 57.6) | 63.4 | (61.4 - 65.4) |
| position 2 | 22.9 | (21.0 - 24.9) | 26.0 | (23.8 - 28.2) | 21.4 | (19.7 - 23.1) |
| position 3 | 9.6 | (8.2 - 10.9) | 10.9 | (9.3 - 12.5) | 9.3 | (8.1 - 10.5) |
| position 4 | 4.0 | (3.1 - 5.0) | 4.2 | (3.2 - 5.2) | 4.1 | (3.2 - 4.9) |
| position 5 | 2.2 | (1.5 - 2.8) | 3.8 | (2.8 - 4.7) | 1.8 | (1.3 - 2.4) |
| **BHIS 2004** |  |  |  |  |  |  |
| position 1 | 62.0 | (60.0 - 64.0) | 51.5 | (49.3 - 53.8) | 65.2 | (63.3 - 67.1) |
| position 2 | 21.2 | (19.4 - 22.9) | 23.9 | (21.9 - 25.8) | 21.3 | (19.6 - 22.9) |
| position 3 | 9.0 | (7.8 - 10.2) | 11.0 | (9.6 - 12.4) | 7.4 | (6.3 - 8.4) |
| position 4 | 3.0 | (2.3 - 3.7) | 4.8 | (3.8 - 5.7) | 3.3 | (2.5 - 4.0) |
| position 5 | 4.8 | (3.9 - 5.7) | 8.8 | (7.6 - 10.1) | 2.9 | (2.2 - 3.6) |
| **BHIS 2008** |  |  |  |  |  |  |
| position 1 | 55.0 | (52.8 - 57.2) | 39.4 | (37.2 - 41.7) | 54.3 | (52.2 - 56.5) |
| position 2 | 22.4 | (20.5 - 24.2) | 21.2 | (19.3 - 23.1) | 19.4 | (17.7 - 21.2) |
| position 3 | 9.9 | (8.5 - 11.2) | 10.8 | (9.3 - 12.2) | 10.1 | (8.8 - 11.5) |
| position 4 | 3.8 | (3.0 - 4.7) | 7.1 | (5.9 - 8.2) | 4.1 | (3.2 - 5.0) |
| position 5 | 8.9 | (7.6 - 10.2) | 21.5 | (19.6 - 23.4) | 12.0 | (10.6 - 13.4) |
| **BHIS 2013** |  |  |  |  |  |  |
| position 1 | 56.9 | (54.5 - 59.3) | 49.4 | (46.8 - 52.0) | 58.6 | (56.5 - 60.8) |
| position 2 | 23.8 | (21.7 - 25.9) | 20.5 | (18.4 - 22.6) | 22.5 | (20.7 - 24.4) |
| position 3 | 9.8 | (8.3 - 11.2) | 12.2 | (10.5 - 13.9) | 8.9 | (7.7 - 10.2) |
| position 4 | 4.9 | (3.9 - 6.0) | 7.2 | (5.8 - 8.5) | 4.3 | (3.4 - 5.2) |
| position 5 | 4.6 | (3.6 - 5.6) | 10.8 | (9.2 - 12.4) | 5.6 | (4.6 - 6.6) |
| **BHIS 2018** |  |  |  |  |  |  |
| position 1 | 53.6 | (51.2 - 56.1) | 48.8 | (46.6 - 51.0) | 54.4 | (52.2 - 56.5) |
| position 2 | 23.5 | (21.4 - 25.6) | 23.6 | (21.8 - 25.5) | 22.9 | (21.1 - 24.8) |
| position 3 | 9.2 | (7.8 - 10.6) | 11.9 | (10.5 - 13.3) | 9.8 | (8.5 - 11.1) |
| position 4 | 2.3 | (1.5 - 3.0) | 4.5 | (3.6 - 5.4) | 4.6 | (3.7 - 5.5) |
| position 5 | 11.4 | (9.9 - 13.0) | 11.1 | (9.8 - 12.5) | 8.3 | (7.1 - 9.5) |
|  |  |  |  |  |  |  |
| difference in probs for position 1 in 2018 versus 1997 | -1.0 | (-4.5 – 2.5) | 2.0 | (-1.3 – 5.3) | 2.0 | (-1.2 – 5.3) |
| difference in probs for position 2 in 2018 versus 1997 | 0.1 | (-2.9 – 3.0) | -1.0 | (-3.9 - 1.8) | -1.3 | (-4.1 – 4.4) |
| difference in probs for position 3 in 2018 versus 1997 | -1.5 | (-3.6 – 0.6) | -2.8 | (-5.1 - -0.6) | -2.8 | (-4.9 - -0.7) |
| difference in probs for position 4 in 2018 versus 1997 | -2.2 | (-3.4 - -0.9) | -1.7 | (-3.2 - -0.2) | -0.5 | (-1.9 – 4.2) |
| difference in probs for position 5 in 2018 versus 1997 | 4.6 | (2.6 – 6.6) | 3.6 | (1.7 – 5.5) | 2.6 | (0.9 – 4.2) |
